# Trends in Alzheimer's Disease and Dementia in the Asian-Pacific Region

**DOI:** 10.1155/2012/171327

**Published:** 2012-12-09

**Authors:** Neelum T. Aggarwal, Manjari Tripathi, Hiroko H. Dodge, Suvarna Alladi, Kaarin J. Anstey

**Affiliations:** ^1^Rush Alzheimer's Disease Center, Rush University Medical Center, Suite 1038 AAC, 600 South Paulina, Chicago, IL 60612, USA; ^2^Department of Neurology, All India Institute of Medical Sciences, New Delhi 110029, India; ^3^Department of Neurology, Oregon Health and Sciences University, Portland, OR 97329-3098, USA; ^4^Department of Neurology, Nizam's Institute of Medical Sciences, Panjagutta, Hyderabad, Andhra Pradesh 500082, India; ^5^The Centre for Research on Ageing, Health and Wellbeing, The Australian National University, Canberra, ACT 0200, Australia

## 1. Introduction

Dementia is an age associated illness with a devastating impact on patients and their families. In the USA, the broader society impact of dementia continues to be overwhelming, due largely to the huge health care and economic burden associated with the disease. Although the projected numbers of those affected, the economic, healthcare, and caregiver costs continue to have a place in US public policy, it is only recently that these issues are beginning to take a center stage in other regions in the world with their aging populations. It has been estimated that 35.6 million people are living with dementia worldwide—a number that is projected to increase to 65.7 million by 2030 and 115.4 million by 2050.

Approximately, 60% of the worlds' population lives in the Asian Pacific region—a home to many different ethnic groups. This issue of the International Journal of Alzheimer's Disease is dedicated to dementia in the Asian and Pacific region and discusses from an Asian-Pacific perspective common themes often noted in the literature from Europe and North America. Themes discussed in this special issue include (1) the prevalence and incidence rates of dementia in Asian countries, (2) the role of biological and genetic risk factors to the development of dementia, (3) characterization of dementia in culturally diverse populations, and (4) activities of daily living functioning and its relation to cognitive functioning.

In this issue, two studies examined the prevalence, incidence, and mortality rates of dementia. H. H. Dodge et al. examined changes in dementia prevalence and the relative prevalence of AD compared to VaD over time using eight large Japanese prevalence studies. Unlike past studies on this topic, the authors thoroughly examined diagnostic criteria used in each study (through contacting original investigators of the most studies), changing age structures as well as regional variability as possible explanations of trends in overall prevalence and ratios of AD to VaD. The study suggests that, in contrast to the USA and some European countries, all-cause dementia prevalence is increasing in Japan. It was inconclusive whether the prevalence of AD as opposed to VaD has been increasing or not, because of variability in diagnostic criteria, regional variability, and gender difference in vascular disease prevalence. This study illustrates the complexity of evaluating prevalence rates of dementia and how knowledge of population trends in risk factors and diagnostic methods influences the interpretation of data. In addition, the authors offer useful suggestions for future epidemiological work on dementia prevalence and incidence in Japan which may be applied to other countries.

In another article, the prevalence rates and mortality of dementia was examined in elderly persons living in Hong Kong. This study suggests that within 30 years the number of people in Hong Kong aged 60 and older will be more than triple; thus, the increase of prevalence of dementia cases in this region will prove to be substantial. The authors also discuss two other important issues related to care for those with dementia: (1) the impact of a declining “oldest old support ratio” and (2) the burden of dementia as measured by the Disability Adjusted Life Years (DALYs) approach. The authors postulate that by using these two metrics, in addition to data regarding the ongoing trends in the region, a successfully long-term care strategy for dementia of the aging population in Hong Kong can be achieved.

## 2. The Role of Biological and Genetic Risk Factors to Dementia

The article by L. N. Kota et al. examined the role of diabetes, APOE e4 carrier state to dementia in a cohort of elderly persons in Southern India. In India, the latest data suggest that the prevalence of dementia cases, predominantly that of Alzheimer's disease (AD), is estimated to increase from 3.7 million people to 6.35 million people by 2025. This staggering increase is accompanied by an ongoing pandemic rise of diabetes in Indians, with current estimates at 40.9 million persons affected. How these two factors are associated with genetic risk factors, namely, APOEe4 carrier status was the focus of this article. Two important findings were presented in this article: (1) the APOE e4 allele appears to be associated with AD in this population—thus replicating findings from previous studies in this population and (2) persons with AD who have diabetes were more likely to be APOE e4 positive compared to those with normal cognition. The authors conclude that although more work needs to be done in this area, lifestyle modification to prevent diabetes, thereby perhaps slowing the prevalence rate of dementia and AD, should be a major focus on public health initiatives in this region.

## 3. Characterizing Dementia in Culturally Diverse Populations

The article by G. Nair et al. provides valuable insights into the characteristics of a memory clinic within a public hospital in India, a country that is expected to have more than 150 million older adults by 2025. The authors describe the challenges of establishing a clinical research based facility that provides services for patients with significant linguistic and cultural diversity. This article provides invaluable information on the methods used to establish research facilities in a developing country and documents the practical aspects such as staff training, outreach, and consensus diagnosis. The memory clinic has implemented the National Alzheimer's Coordinating Center (NACC) standardized Uniform Data Set (UDS) evaluation and will provide an invaluable resource for research, both within India and internationally. Although the sample is not representative, presentation of some descriptive data on the rates of diagnosis and demographic characteristics of diagnostic groups shows that dementia diagnosis, particularly Alzheimer's disease, is occurring at relative young ages in this community. Such information over time may provide important insights into culturally specific risk factors.

A second article examined another area of focus in cognitive research in diverse populations—the development and utilization of linguistically and culturally validated, cognitive screening tests. L. Zheng et al. developed a Chinese version of the Montreal Cognitive Assessment (MOCA)—the MOCA-Chinese Los Angeles (MOCA-ChLA)— that can be used with Cantonese or Mandarin speakers. The MOCA was carefully translated and culturally adapted for use in the two languages (as well as in Taiwanese) and administered to over 1,000 ethnic Chinese elderly residing in the Los Angeles area who were taking part in a population-based Chinese-American Eye Study (CHES). The MOCA-ChLA was found to be free of floor and ceiling effects, scores were not influenced by gender, and Mandarin and Cantonese speaking subgroups performed similarly. As the authors noted, further studies are warranted to determine cutpoints for optimal sensitivity, specificity for the diagnosis of MCI and dementia, and refine adjustment scores for individuals with low education; so that the MOCA-ChLA can be used for a screening tool for identifying MCI and dementia in other communities.

## 4. Activities of Daily Living and Dementia

As dementia becomes more prominent in both Eastern and Western cultures, it is well known that maintaining activities of daily living (ADL) becomes a major focus for care. Past studies have shown that person-centered care, where care is targeted to encourage, support, and maintain patients' hobbies and leisure activities (in addition to supporting medical and basic IADL needs), improves the quality of life (QOL) of patients and reduces behavioral psychological symptoms of dementia (BPSD). Yet, maintaining patients' advanced activities of daily living (AADL: e.g., participation in meetings, socializing with others, taking a walk, reading a newspaper, watching TV, etc.) and leisure activities (e.g., travelling, care of a pet, gardening, etc.) is often ignored in public social services. H. Takechi et al. examined types, frequency, and support needs on AADL and leisure activities by interviewing 39 pairs of early-to-mid stage dementia patients and their family caregivers. This was a time-consuming study because a large variability in patients' leisure activities required in-depth interviews with patients and caregivers, using open-ended questions for some categories. Despite the difficulty, the authors were able to outline potential domains and types of cares required for person-centered care. This type of study provides support for more studies in this area, as it can lead to a better quality of life for those affected by dementia and also their caregivers.

## 5. Summary

In this special issue, we highlight several important themes emerging from the Asia-Pacific region as they relate to dementia research. The encompassing message is that this region, with its rapidly growing population of persons with dementia, will place a heavy societal and economic burden to all countries in the region. Studies on the possible inter-ethnic differences in prevalence and incidence of dementia, risk factors and protective factors, and treatment for intervention strategies need to be employed on an international level.

*Neelum T. Aggarwal* *Neelum T. Aggarwal* *Manjari Tripathi* *Manjari Tripathi* *Hiroko H. Dodge* *Hiroko H. Dodge* *Suvarna Alladi* *Suvarna Alladi* *Kaarin J. Anstey* *Kaarin J. Anstey*

